# Effects of *ADIPOQ* polymorphisms on individual susceptibility to coronary artery disease: a meta-analysis

**DOI:** 10.1080/21623945.2019.1595270

**Published:** 2019-03-24

**Authors:** Zhiyuan Wang, Jinglan Diao, Xin Yue, Jingquan Zhong

**Affiliations:** a Department of Cardiology, Yuncheng County People’s Hospital, Heze, China; b Department of Cardiology, Qilu Hospital of Shandong University, Jinan, China

**Keywords:** Adiponectin (*ADIPOQ*), genetic polymorphisms, coronary artery disease (CAD), meta-analysis

## Abstract

Whether adiponectin (*ADIPOQ*) polymorphisms affect individual susceptibility to coronary artery disease (CAD) remains controversial. Therefore, we performed this meta-analysis to better analyse associations between *ADIPOQ* polymorphisms and CAD. PubMed, Web of Science, Embase and CNKI were searched for eligible studies. Odds ratios (ORs) and 95% confidence intervals (CIs) were calculated. Totally, 51 studies were eligible for analyses. In overall analyses, significant associations with the susceptibility to CAD were detected for rs266729 (overdominant model: *p*= 0.03, OR = 1.11, 95% CI 1.01–1.22), rs822395 (recessive model: *p*= 0.007, OR = 1.21, 95% CI 1.05–1.40) and rs2241766 (dominant model: *p*= 0.0009, OR = 0.82, 95% CI 0.73–0.92; recessive model: *p*= 0.04, OR = 1.29, 95% CI 1.02–1.64; allele model: *p*< 0.0001, OR = 0.80, 95% CI 0.73–0.88) polymorphisms. Further subgroup analyses by ethnicity revealed that rs1501299 polymorphism was significantly associated with the susceptibility to CAD in East Asians, while rs2241766 polymorphism was significantly associated with the susceptibility to CAD in Caucasians, East Asians and South Asians. In summary, our findings indicated that rs266729, rs822395, rs1501299 and rs2241766 polymorphisms were all significantly associated with the susceptibility to CAD in certain populations.

## Introduction

Coronary artery disease (CAD) is the leading cause of death and disability worldwide [1,2]. So far, the exact pathogenesis of CAD is still unclear. Nevertheless, plenty of evidence supported that genetic factors may play a crucial part in its development. First, family clustering of CAD was observed extensively, and past twin studies proved that the heredity grade of CAD was over 50% [3,4]. Second, numerous genetic variants were found to be associated with an increased susceptibility to CAD by previous genetic association studies, and screening of common causal variants was also proved to be an efficient way to predict the individual risk of developing CAD [5,6]. Overall, these findings jointly supported that genetic predisposition to CAD is important for its occurrence and development.

Adiponectin (ADIPOQ), a multifunctional adipocytokine that is predominantly secreted by adipocytes, plays a central role in regulating energy and material metabolism [7]. Previous studies showed that adiponectin has both anti-atherogenic and anti-inflammatory properties [8,9]. Furthermore, the expression level of adiponectin was also significantly decreased in patients with CAD [10,11]. In summary, these pieces of evidence jointly suggested that adiponectin might exert favourable protection effects against CAD. Therefore, functional *ADIPOQ* genetic polymorphisms, which may alter the expression level of adiponectin, may also affect individual susceptibility to CAD. So far, several studies already tried to investigate associations between *ADIPOQ* polymorphisms and CAD, but the results of these studies were controversial, especially when they were conducted in different populations [12–19]. Previous studies failed to reach a consensus regarding associations between *ADIPOQ* polymorphisms and CAD partially because of their relatively small sample sizes. Thus, we performed the present meta-analysis to explore the relationship between *ADIPOQ* polymorphisms and CAD in a larger pooled sample size. Additionally, we also aimed to elucidate the potential effects of ethnic background on associations between *ADIPOQ* polymorphisms and CAD.

## Materials and methods

### Literature search and inclusion criteria

The current meta-analysis followed the Preferred Reporting Items for Systematic Reviews and Meta-Analyses (PRISMA) checklist [20]. PubMed, Web of Science, Embase and China National Knowledge Infrastructure (CNKI) were searched for potentially eligible articles using the combination of following terms: (adiponectin OR ADIPOQ) AND (polymorphism OR variant OR mutation OR genotype OR allele) AND (coronary heart disease OR coronary artery disease OR angina pectoris OR acute coronary syndrome OR myocardial infarction). We also reviewed the reference lists of all retrieved articles to identify other potentially eligible studies. The initial search was conducted in July 2018 and the latest update was performed in December 2018.

To test the research hypothesis of this meta-analysis, included studies must satisfy the following criteria: (1) case–control study on associations between *ADIPOQ* polymorphisms and CAD; (2) provide genotypic and/or allelic frequency of investigated *ADIPOQ* polymorphisms; and (3) full text in English or Chinese available. Studies were excluded if one of the following criteria was fulfilled: (1) not relevant to *ADIPOQ* polymorphisms and CAD; (2) case reports or case series; and (3) abstracts, reviews, comments, letters and conference presentations. In the case of duplicate reports by the same authors, we only included the most recent study for analyses.

### Data extraction and quality assessment

We extracted the following information from eligible studies: (a) name of the first author; (b) year of publication; (c) country and ethnicity of participants; (d) sample size; and (e) genotypic distributions of *ADIPOQ* polymorphisms in cases and controls. The probability value (*p* value) of Hardy–Weinberg equilibrium (HWE) was also calculated.

We used the Newcastle–Ottawa scale (NOS) to evaluate the quality of eligible studies [21]. The NOS has a score range of 0 to 9, and studies with a score of more than 7 were thought to be of high quality.

Two reviewers conducted data extraction and quality assessment independently. When necessary, we wrote to the corresponding authors for extra information. Any disagreement between two reviewers was solved by discussion until a consensus was reached.

### Statistical analyses

In the current study, we performed statistical analyses by using Review Manager Version 5.3.3. We calculated ORs and 95% CIs to estimate potential associations between *ADIPOQ* polymorphisms and CAD in dominant, recessive, overdominant and allele models, and statistical significances of pooled analyses were determined by the *Z* test, with a *p* value of 0.05 or less was defined as statistically significant. All investigated *ADIPOQ* polymorphisms contain a major allele (M) and a minor allele (m), and the definitions of all genetic comparisons were as follows: dominant comparison is defined as MM versus Mm + mm, recessive comparison is defined as mm vs. MM +Mm, overdominant comparison is defined as Mm versus MM + mm, and the allele comparison is defined as M versus m. Between-study heterogeneities were evaluated by *I*^2^ statistic. Random-effect models would be used for analyses if *I*^2^ was greater than 50% (Der Simonian–Laird method). Otherwise, analyses would be conducted with fixed-effect models (Mantel–Haenszel method). Subgroup analyses were subsequently carried out by ethnicity and type of disease. Stabilities of synthetic results were tested in sensitivity analyses. Publication biases were assessed by funnel plots.

## Results

### Characteristics of included studies

**Figure 1.**
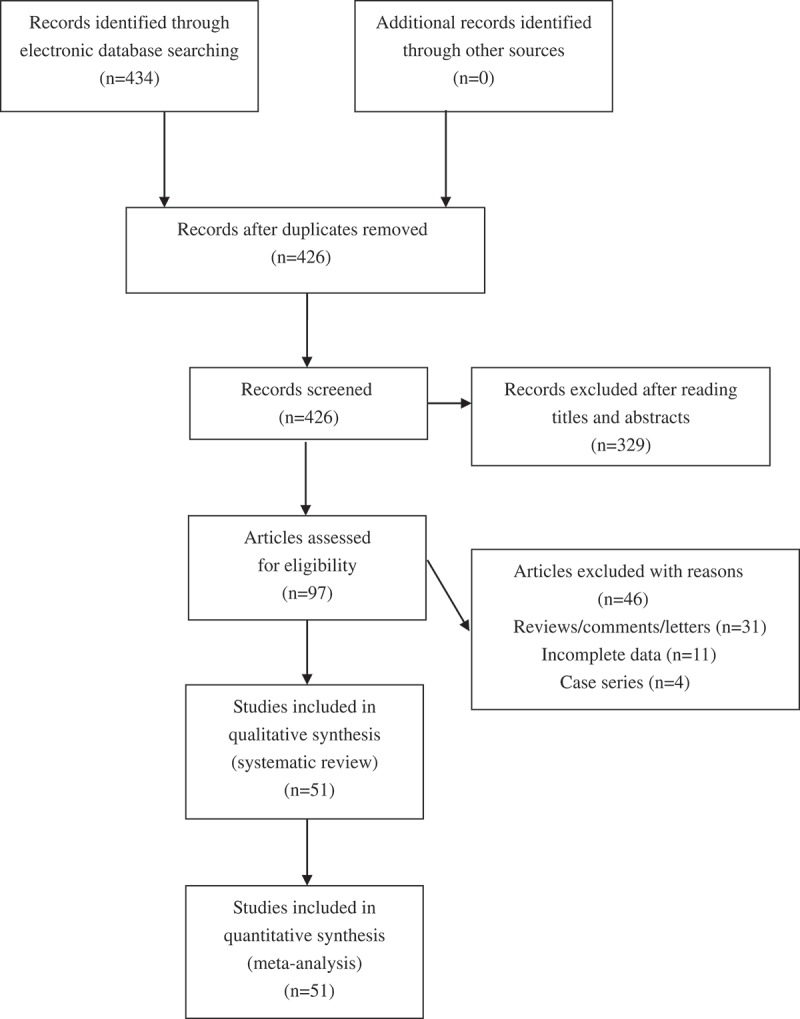
Flowchart of study selection for the present study.

We found 434 potentially relevant articles. Among these articles, totally 51 eligible studies were finally included for synthetic analyses (see Figure 1). The NOS score of eligible articles ranged from 7 to 8, which indicated that all the included studies were of high quality. Baseline characteristics of the included studies are summarized in Table 1.

**Table 1.** The characteristics of included studies.

First author, y	Country	Ethnicity	Type of disease	Sample size	Genotype distribution		*p*-Value for HWE	NOS score
					Case–controls			
**rs266729 G/C**					CC/CG/GG			
Cheung 2014	Hong Kong	East Asian	CAD	184/2007	111/65/8	1148/729/130	0.327	7
Chiodini 2010	Italy	Caucasian	MI	1002/503	583/353/66	321/160/22	0.717	7
De Caterina 2011	Italy	Caucasian	MI	1855/1855	1076/671/108	1063/684/108	0.883	7
Du 2016	China	East Asian	CAD	493/304	278/175/40	219/73/12	0.069	8
Gable 2007	UK	Caucasian	MI	530/564	278/217/35	329/197/38	0.254	8
Hegener 2006	USA	Mixed	MI	340/342	197/123/20	188/134/20	0.543	8
Lacquemant 2004	UK	Caucasian	CAD	161/313	89/65/7	174/118/21	0.870	7
Oguri 2009	Japan	East Asian	MI	773/1114	397/336/40	675/379/60	0.478	7
Persson 2010	Sweden	Caucasian	MI	244/244	127/100/17	130/101/13	0.241	8
Prior 2009	UK	Caucasian	CAD	155/609	89/56/10	335/242/32	0.165	8
Prior 2011	UK	Caucasian	CAD	85/298	46/38/1	158/114/26	0.406	8
Rodr´ıguez-Rodr´ıguez 2011	Spain	Caucasian	CAD	119/555	67/46/6	327/188/40	0.076	7
Zhang 2015	China	East Asian	CAD	561/412	305/228/28	212/172/28	0.383	8
Zhang 2018	China	East Asian	CAD	717/612	345/306/66	301/253/58	0.648	8
Zhao 2018	China	East Asian	CAD	1044/1349	590/385/69	774/498/77	0.791	8
Zhong 2010	China	East Asian	CAD	198/237	110/72/16	146/76/15	0.239	8
**rs822395 A/C**					AA/AC/CC			
Cheung 2014	Hong Kong	East Asian	CAD	184/2009	130/53/1	1441/527/41	0.371	7
De Caterina 2011	Italy	Caucasian	MI	1855/1854	848/811/196	867/806/181	0.750	7
Lacquemant 2004	UK	Caucasian	CAD	162/311	75/69/18	138/141/32	0.647	7
Pischon 200	USA	Mixed	CAD	496/989	223/208/65	450/467/72	0.001	7
Qi 2005	USA	Mixed	CAD	234/626	104/101/29	270/280/76	0.795	7
Zhang 2015	China	East Asian	CAD	535/396	408/119/8	274/114/8	0.328	8
Zhang 2018	China	East Asian	CAD	717/612	295/307/115	252/281/79	0.962	8
Zhong 2010	China	East Asian	CAD	198/237	143/48/7	175/59/3	0.424	8
**rs1501299 G/T**					GG/GT/TT			
Al-Daghri 2011	Saudi Arabia	South Asian	CAD	123/297	47/57/19	111/142/44	0.897	7
Ambroziak 2018	Poland	Caucasian	MI	188/153	88/72/28	84/59/10	0.933	7
Antonopoulos 2013	Greece	Caucasian	CAD	462/132	220/212/30	66/50/16	0.184	8
Bacci 2004	Italy	Caucasian	CAD	142/234	70/65/7	118/88/28	0.073	7
Boumaiza 2011	Tunisia	Caucasian	CAD	213/108	105/84/23	45/41/18	0.115	8
Chen 2011	China	East Asian	CAD	93/102	54/33/6	61/38/3	0.307	7
Cheung 2014	Hong Kong	East Asian	CAD	182/2010	88/75/19	1103/759/148	0.270	7
Chiodini 2010	Italy	Caucasian	MI	1002/503	530/392/80	239/198/66	0.016	7
De Caterina 2011	Italy	Caucasian	MI	1833/1821	926/746/161	906/767/148	0.419	7
Esteghamati 2012	Iran	South Asian	CAD	114/127	76/30/8	63/47/17	0.095	7
Filippi 2005	Italy	Caucasian	CAD	580/466	287/241/52	266/167/33	0.338	8
Gable 2007	UK	Caucasian	MI	504/557	266/216/22	289/225/43	0.931	8
Ghazouani 2018	Tunisia	Caucasian	CAD	277/269	143/93/41	138/88/43	<0.001	8
Gui 2012	China	East Asian	CAD	410/431	172/185/53	239/154/38	0.072	8
Hegener 2006	USA	Mixed	MI	341/341	183/134/24	181/143/17	0.093	8
Jung 2006	Korea	East Asian	CAD	88/68	38/43/7	31/32/5	0.399	7
Katakami 2012	Japan	East Asian	MI	213/2424	129/71/13	1229/976/219	0.209	7
Lacquemant 2004	UK	Caucasian	CAD	161/309	82/66/13	169/115/25	0.387	7
Li 2018	China	East Asian	CAD	201/141	67/107/27	64/53/24	0.030	8
Liang 2011	China	East Asian	MI	78/84	30/43/5	48/30/6	0.663	7
Liang 2017	China	East Asian	CAD	960/962	490/388/82	617/300/45	0.275	8
Mohammadzadeh 2016	Iran	South Asian	CAD	100/100	38/55/7	56/42/2	0.063	7
Ohashi 2004	Japan	East Asian	CAD	383/368	185/164/34	190/149/29	0.977	8
Oliveira 2012	Brazil	Mixed	CAD	450/153	209/197/44	62/68/23	0.542	7
Pischon 2007	USA	Mixed	CAD	491/988	266/182/43	485/416/87	0.869	7
Qi 2005	USA	Mixed	CAD	228/594	105/111/12	293/249/52	0.930	7
Rizk 2012	Qatar	South Asian	ACS	142/121	58/64/20	46/59/16	0.667	7
Rodr´ıguez-Rodr´ıguez 2011	Spain	Caucasian	CAD	119/555	69/44/6	287/224/44	0.975	7
Wu 2013	China	East Asian	CAD	188/200	67/108/13	92/90/18	0.545	7
Zhang 2015	China	East Asian	CAD	561/412	309/209/43	214/170/28	0.459	8
Zhang 2018	China	East Asian	CAD	717/612	583/126/8	471/131/10	0.798	8
**rs2241766 T/G**					TT/TG/GG			
Al-Daghri 2011	Saudi Arabia	South Asian	CAD	122/298	77/35/10	220/72/6	0.969	7
Antonopoulos 2013	Greece	Caucasian	CAD	462/132	359/97/6	99/29/4	0.309	8
Bacci 2004	Italy	Caucasian	CAD	130/220	90/35/5	149/60/11	0.135	7
Boumaiza 2011	Tunisia	Caucasian	CAD	212/104	145/57/10	75/24/5	0.111	8
Chang 2009	Taiwan	East Asian	CAD	600/687	316/238/46	309/399/79	0.606	7
Chen 2011	China	East Asian	CAD	93/102	68/19/6	59/35/8	0.391	7
Cheung 2014	Hong Kong	East Asian	CAD	184/2012	89/83/12	1007/822/183	0.413	7
Chiodini 2010	Italy	Caucasian	MI	1002/503	679/304/19	359/126/18	0.102	7
Di 2011	China	East Asian	CAD	196/124	91/85/20	65/50/9	0.884	7
Du 2016	China	East Asian	CAD	493/304	253/190/50	185/97/22	0.069	8
Esteghamati 2012	Iran	South Asian	CAD	114/127	48/41/25	68/46/13	0.222	7
Foucan 2010	French West Indies	African	CAD	57/159	NA	NA	NA	7
Gable 2007	UK	Caucasian	MI	526/563	360/154/12	384/168/11	0.280	8
Ghazouani 2018	Tunisia	Caucasian	CAD	277/269	181/74/22	182/70/17	0.007	8
Hegener 2006	USA	Mixed	MI	341/341	241/95/5	252/80/9	0.389	8
Jin 2009	China	East Asian	CAD	110/73	53/48/9	50/20/3	0.584	8
Jung 2006	Korea	East Asian	CAD	88/68	41/40/7	34/30/4	0.431	7
Lacquemant 2004	UK	Caucasian	CAD	162/315	109/48/5	249/57/9	0.015	7
Li 2011	China	East Asian	CAD	118/97	51/46/21	54/31/12	0.036	8
Liang 2017	China	East Asian	CAD	960/982	471/382/107	608/308/46	0.387	8
Luo 2010	China	East Asian	CAD	221/100	100/99/22	50/41/9	0.886	7
Mofarrah 2016	Iran	South Asian	CAD	152/72	82/35/35	56/13/3	0.072	8
Mohammadzadeh 2016	Iran	South Asian	CAD	100/100	75/24/1	65/31/4	0.900	7
Nan 2012	China	East Asian	CAD	213/467	115/84/14	237/191/39	0.953	8
Oliveira 2012	Brazil	Mixed	CAD	450/153	323/114/13	117/33/3	0.708	7
Pischon 2007	USA	Mixed	CAD	482/979	374/102/6	759/202/18	0.290	7
Qi 2005	USA	Mixed	CAD	219/599	NA	NA	NA	7
Rizk 2012	Qatar	South Asian	ACS	142/122	62/42/38	56/49/17	0.245	7
Sabouri 2011	Iran	South Asian	CAD	329/241	253/74/2	205/35/1	0.703	7
Xu 2010	China	East Asian	CAD	153/73	78/65/10	50/20/3	0.584	8
Zhang 2011	China	East Asian	CAD	149/167	63/60/26	97/50/20	0.002	7
Zhang 2015	China	East Asian	CAD	561/412	276/235/50	224/164/24	0.399	8
Zhang 2018	China	East Asian	CAD	717/612	500/184/33	456/149/7	0.177	8
**rs17300539 G/A**					GG/GA/AA			
Ambroziak 2018	Poland	Caucasian	MI	193/153	169/23/1	130/23/0	0.315	7
Chiodini 2010	Italy	Caucasian	MI	1002/503	827/165/10	414/87/2	0.252	7
Gable 2007	UK	Caucasian	MI	529/568	446/78/5	458/107/3	0.220	8
Oliveira 2012	Brazil	Mixed	CAD	449/153	388/56/5	131/22/0	0.338	7
Zhang 2018	China	East Asian	CAD	717/612	614/100/3	542/67/3	0.553	8

CAD: coronary artery disease; MI: myocardial infarction; ACS: acute coronary syndrome; HWE:Hardy-Weinberg Hardy–Weinberg equilibrium; NOS: Newcastle-Ottawa Newcastle–Ottawa scale; NA: not available.

### Overall and subgroup analyses

Results of overall and subgroup analyses are summarized in Table 2. To be brief, significant associations with the susceptibility to CAD were detected for rs266729 (overdominant model: *p* = 0.03, odds ratio [OR] = 1.11, 95% confidence interval [CI] 1.01–1.22), rs822395 (recessive model: *p* = 0.007, OR = 1.21, 95% CI 1.05–1.40) and rs2241766 (dominant model: *p* = 0.0009, OR = 0.82, 95% CI 0.73–0.92; recessive model: *p* = 0.04, OR = 1.29, 95% CI 1.02–1.64; allele model: *p* < 0.0001, OR = 0.80, 95% CI 0.73–0.88) polymorphisms in overall analyses. Further subgroup analyses by ethnicity revealed that rs1501299 polymorphism was significantly associated with the susceptibility to CAD in East Asians, while rs2241766 polymorphism was significantly associated with the susceptibility to CAD in Caucasians, East Asians and South Asians. No any other positive results were observed in overall and subgroup analyses (see Table 2 and supplementary Figure 1).

**Table 2.** Results of overall and subgroup analyses for *ADIPOQ* polymorphisms and CAD.

Population	Sample size	Dominant comparison	Recessive comparison	Overdominant comparison	Allele comparison
		*p* Value OR (95% CI) *I*^2^ statistic	*p* Value OR (95% CI) *I*^2^ statistic	*p* Value OR (95% CI) *I*^2^ statistic	*p* Value OR (95% CI) *I*^2^ statistic
**rs266729 C/G**		CC vs. CG + GG	GG vs. CC + CG	CG vs. CC + GG	C vs. G
Overall	8461/11,318	0.06 0.90 (0.81–1.01) 62%	0.69 1.03 (0.91–1.16) 20%	**0.03 1.11 (1.01–1.22**) 52%	0.20 0.94 (0.86–1.03) 65%
Caucasian	4151/4941	0.19 0.94 (0.86–1.03) 6%	0.93 1.01 (0.84–1.21) 28%	0.20 1.06 (0.97–1.16) 8%	0.40 0.97 (0.90–1.04) 36%
East Asian	3970/6035	0.12 0.85 (0.70–1.04) 79%	0.64 1.04 (0.88–1.24) 34%	0.09 1.16 (0.98–1.38) 71%	0.16 0.89 (0.76–1.05) 79%
MI	4744/4622	0.11 0.87 (0.74–1.03) 70%	0.47 1.07 (0.90–1.27) 0%	0.18 1.13 (0.95–1.34) 71%	0.08 0.90 (0.80–1.01) 60%
**rs822395 A/C**		AA vs. AC + CC	CC vs. AA + AC	AC vs. AA + CC	A vs. C
Overall	4381/7034	0.83 1.01 (0.93–1.10) 0%	**0.007 1.21 (1.05–1.40)** 46%	0.07 0.93 (0.85–1.01) 14%	0.30 0.97 (0.91–1.03) 27%
Caucasian	2017/2165	0.63 0.97 (0.86–1.10) 0%	0.39 1.09 (0.89–1.34) 0%	0.97 1.00 (0.88–1.13) 0%	0.45 0.97 (0.88–1.06) 0%
East Asian	1634/3254	0.36 1.07 (0.93–1.24) 42%	0.20 1.20 (0.91–1.59) 39%	0.11 0.89 (0.77–1.03) 33%	0.75 1.03 (0.85–1.25) 55%
**rs1501299 G/T**		GG vs. GT + TT	TT vs. GG + GT	GT vs. GG + TT	G vs. T
Overall	11,544/15,642	0.30 0.94 (0.84–1.05) 73%	0.42 0.94 (0.80–1.10) 57%	0.08 1.09 (0.99–1.19) 60%	0.71 0.98 (0.90–1.08) 76%
Caucasian	5481/5107	0.82 1.01 (0.93–1.09) 39%	0.12 0.80 (0.61–1.06) 67%	0.29 1.04 (0.96–1.13) 2%	0.47 1.04 (0.93–1.17) 64%
East Asian	4074/7814	0.08 0.82 (0.66–1.03) 82%	**0.03 1.20 (1.02–1.42)** 40%	0.10 1.18 (0.97–1.43) 76%	0.14 0.88 (0.74–1.04) 80%
South Asian	479/645	0.88 1.04 (0.61–1.77) 78%	0.97 0.99 (0.68–1.45) 42%	0.79 0.95 (0.65–1.38) 55%	0.90 1.03 (0.68–1.56) 80%
MI	4159/5883	0.67 1.04 (0.87–1.23) 65%	0.63 0.91 (0.63–1.32) 74%	0.42 0.96 (0.88–1.05) 47%	0.71 1.03 (0.88–1.21) 75%
**rs2241766 T/G**		TT vs. TG + GG	GG vs. TT + TG	TG vs. TT + GG	T vs. G
Overall	10,135/11,577	**0.0009 0.82 (0.73–0.92)** 67%	**0.04 1.29 (1.02–1.64)** 63%	0.08 1.12 (0.99–1.27) 71%	**<0.0001 0.80 (0.73–0.88)** 67%
Caucasian	2771/2106	0.09 0.89 (0.79–1.02) 27%	0.39 0.87 (0.62–1.20) 0%	**0.04 1.15 (1.01–1.32)** 33%	0.24 0.93 (0.84–1.05) 20%
East Asian	4856/6280	**0.02 0.80 (0.66–0.96)** 77%	0.06 1.35 (0.99–1.84) 68%	0.30 1.12 (0.90–1.40) 83%	**0.0006 0.80 (0.71–0.91)** 66%
South Asian	959/960	**0.04 0.69 (0.48–0.99)** 66%	**<0.0001 2.67 (1.82–3.91)** 39%	0.76 1.05 (0.76–1.46) 56%	**0.01 0.64 (0.45–0.91)** 76%
MI	1869/1407	0.19 0.90 (0.77–1.05) 0%	0.11 0.68 (0.43–1.09) 18%	0.06 1.16 (0.99–1.36) 30%	0.48 0.95 (0.83–1.09) 0%
**rs17300539 A/G**		AA vs. AG + GG	GG vs. AA + AG	AG vs. AA + GG	A vs. G
Overall	2890/1989	0.73 1.03 (0.88–1.21) 27%	0.12 1.86 (0.85–4.10) 0%	0.46 0.94 (0.80–1.11) 40%	0.89 1.01 (0.87–1.18) 9%
Caucasian	1724/1224	0.19 1.14 (0.94–1.39) 0%	0.12 2.17 (0.81–5.82) 0%	0.09 0.84 (0.69–1.03) 0%	0.37 1.09 (0.90–1.31) 0%
MI	1724/1224	0.19 1.14 (0.94–1.39) 0%	0.12 2.17 (0.81–5.82) 0%	0.09 0.84 (0.69–1.03) 0%	0.37 1.09 (0.90–1.31) 0%

OR: odds ratio; CI: confidence interval; NA: not available; CAD: coronary artery disease; MI: myocardial infarction. The values in bold represent there are statistically significant differences between cases and controls.

### Sensitivity analyses

We performed sensitivity analyses by excluding studies that deviated from HWE. No alterations of results were detected in sensitivity analyses, which suggested that our findings were statistically reliable.

### Publication biases

Publication biases were evaluated with funnel plots. We did not find obvious asymmetry of funnel plots in any comparisons, which indicated that our findings were unlikely to be impacted by severe publication biases (see supplementary Figure 2).

## Discussion

To the best of our knowledge, this is so far the most comprehensive meta-analysis on associations between *ADIPOQ* polymorphisms and CAD, and our pooled analyses demonstrated that rs266729, rs822395, rs1501299 and rs2241766 polymorphisms were all significantly correlated with the susceptibility to CAD in certain populations.

There are several points that need to be addressed about this meta-analysis. First, previous experimental studies showed that mutant alleles of investigated polymorphisms were correlated with decreased adiponectin generation, which may partially explain our positive findings [12–19]. Second, it is also notable that the trends of associations in different ethnicities were not always consistent, and this may be attributed to ethnic differences in genotypic distributions of investigated polymorphisms. However, it is also possible that these inconsistent findings may have resulted from a complex interaction of both genetic and environmental factors. Third, the pathogenic mechanism of CAD is highly complex, and hence, it is unlikely that a single genetic polymorphism could significantly contribute to its development. As a result, to better illustrate potential associations of certain genetic polymorphisms with CAD, we strongly recommend further studies to perform haplotype analyses and explore potential gene–gene interactions.

Some limitations of this meta-analysis should also be noted when interpreting our findings. First, our pooled analyses were based on unadjusted estimations due to lack of raw data, and we have to admit that failure to perform further adjusted analyses may impact the reliability of our findings [22,23]. Second, since our pooled analyses were based on case–control studies, despite our positive findings, future prospective studies are still needed to examine whether there is a direct causal relationship between *ADIPOQ* polymorphisms and CAD [24,25]. Third, associations between *ADIPOQ* polymorphisms and CAD may also be modified by gene–gene and gene–environmental interactions. However, most studies did not consider these potential interactions, which impeded us to conduct relevant analyses [26,27]. Considering the above-mentioned limitations, our findings should be interpreted with caution.

In conclusion, our meta-analysis suggested that rs266729, rs822395, rs1501299 and rs2241766 polymorphisms were all significantly correlated with the susceptibility to CAD in certain populations. However, further well-designed studies are still warranted to confirm our findings.
