# Supplementary material for: Effects of ADIPOQ polymorphisms on individual susceptibility to coronary artery disease: a meta-analysis
Source: Adipocyte. 2019 Mar 24;8(1):137–43. doi: 10.1080/21623945.2019.1595270 (PMC6768194; doi:10.1080/21623945.2019.1595270)
Supplement: Supplemental Material [file kadi-08-01-1595270-s001.zip › Supplementary Figure 1.docx]

Forest plots of investigated polymorphisms


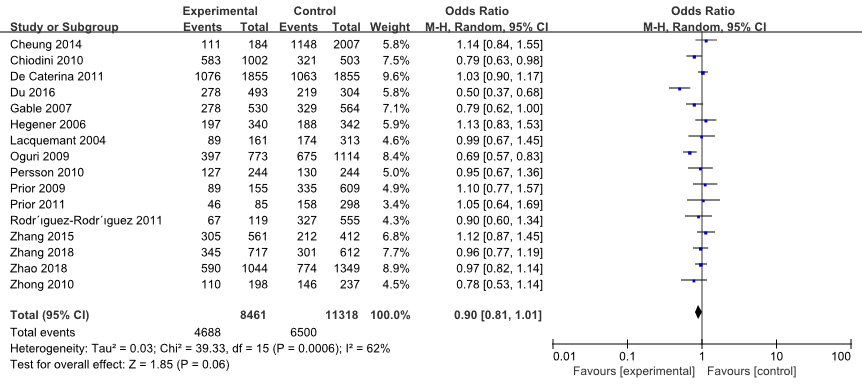


Forest plot of **rs266729** polymorphism and CAD under dominant comparison


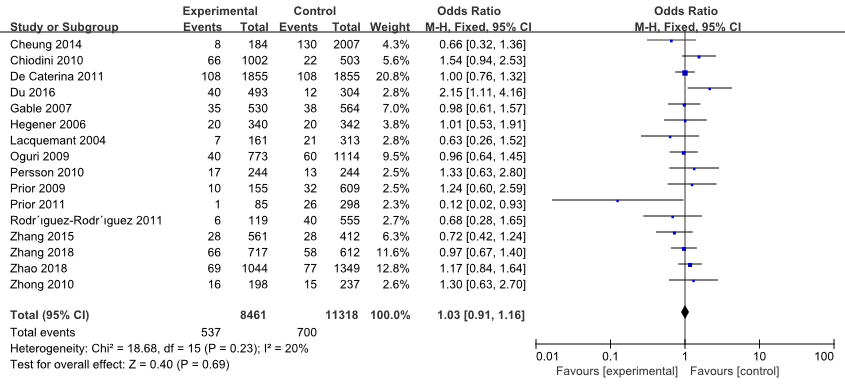


Forest plot of **rs266729** polymorphism and CAD under recessive comparison


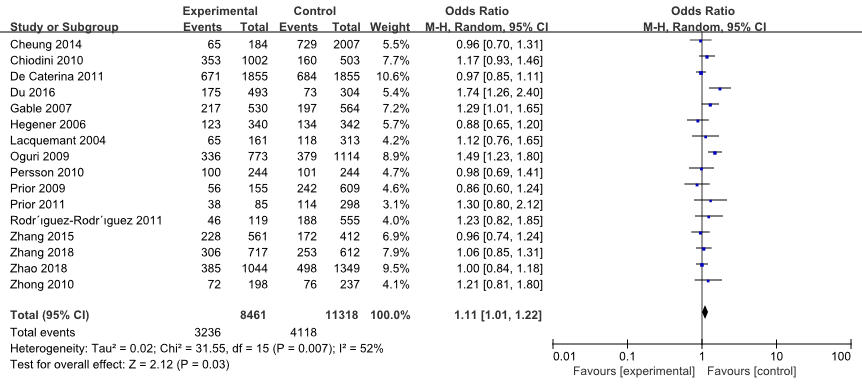


Forest plot of **rs266729** polymorphism and CAD under over-dominant comparison


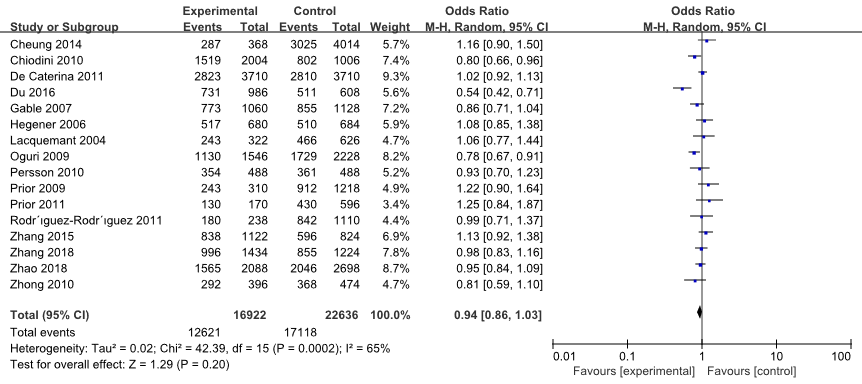


Forest plot of **rs266729** polymorphism and CAD under allele comparison


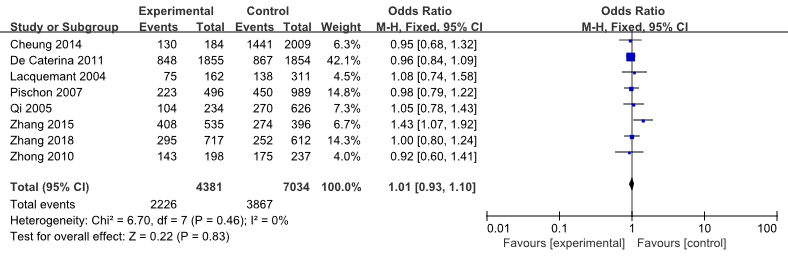


Forest plot of **rs822395** polymorphism and CAD under dominant comparison


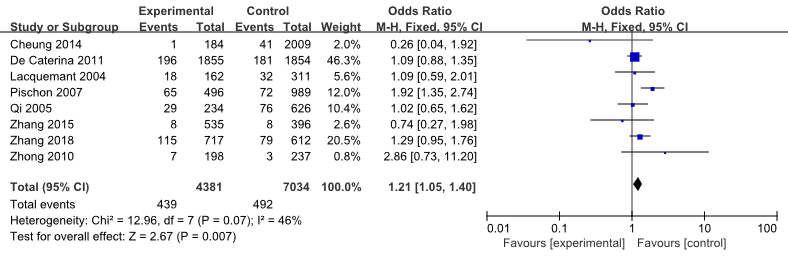


Forest plot of **rs822395** polymorphism and CAD under recessive comparison


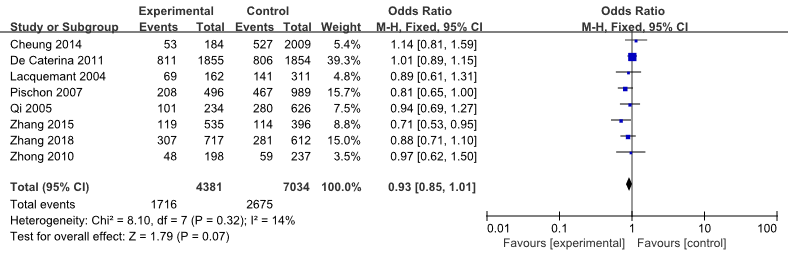


Forest plot of **rs822395** polymorphism and CAD under over-dominant comparison


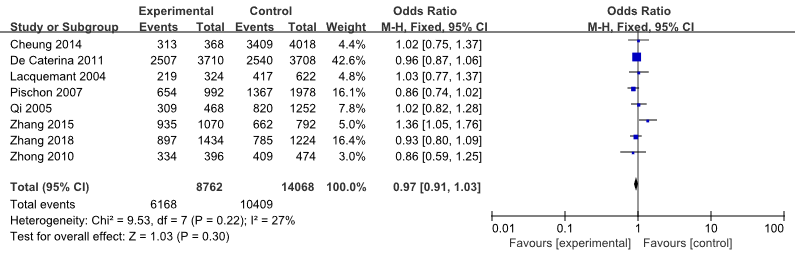


Forest plot of **rs822395** polymorphism and CAD under allele comparison


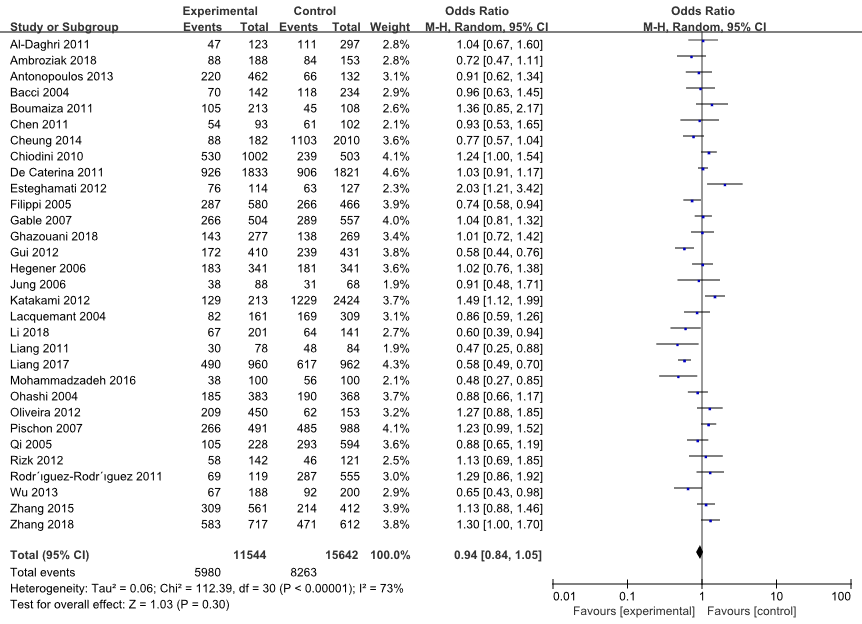


Forest plot of **rs1501299** polymorphism and CAD under dominant comparison

**
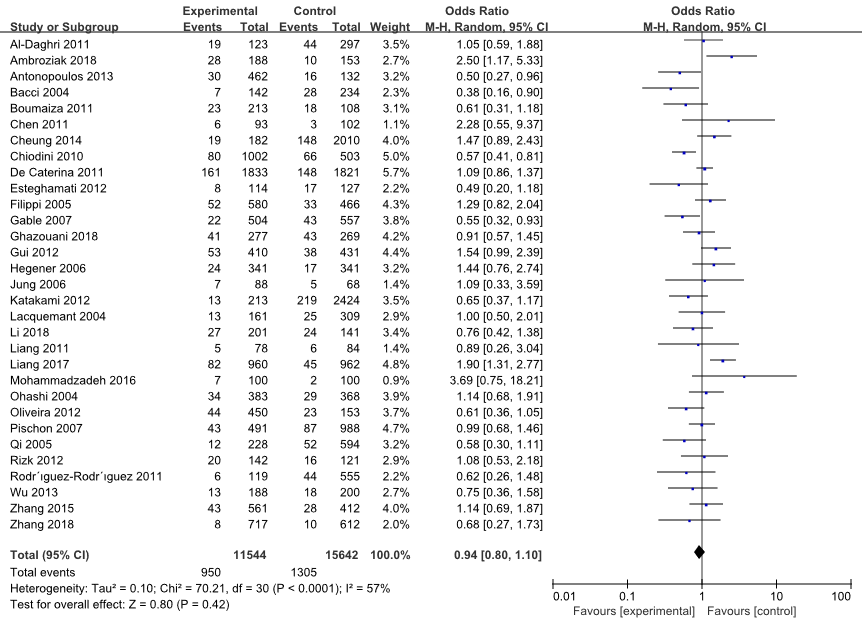
**

Forest plot of **rs1501299** polymorphism and CAD under recessive comparison

**
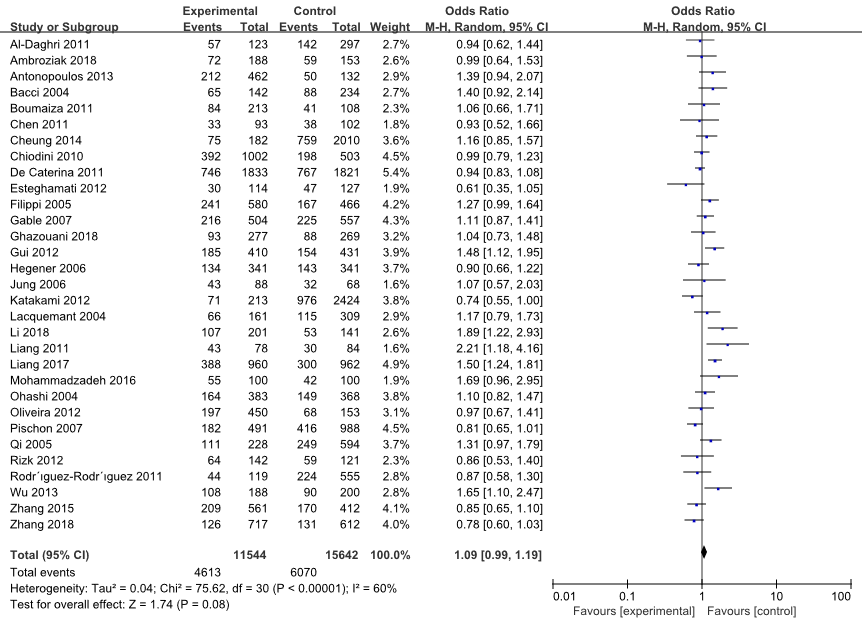
**

Forest plot of **rs1501299** polymorphism and CAD under over-dominant comparison


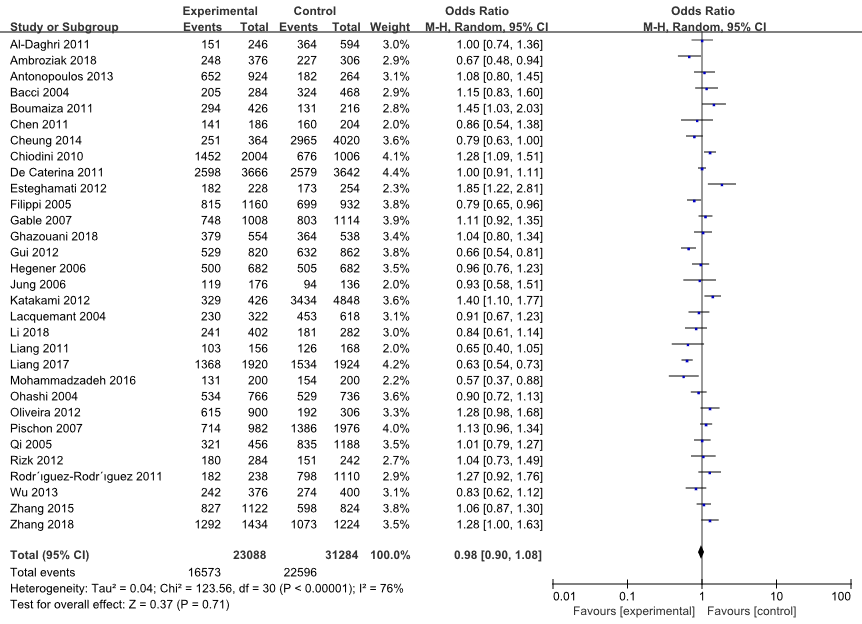


Forest plot of **rs1501299** polymorphism and CAD under allele comparison


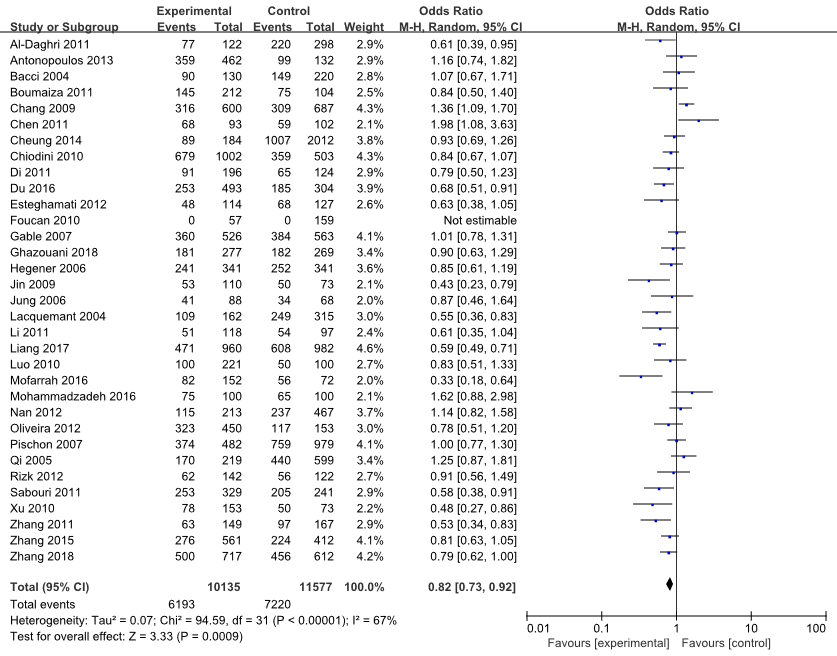


Forest plot of **rs2241766** polymorphism and CAD under dominant comparison

**
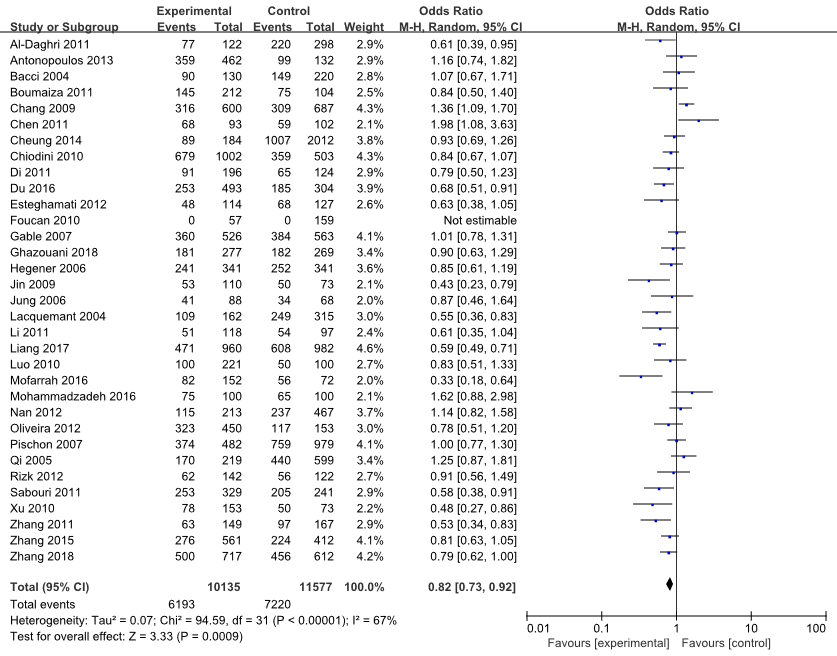
**

Forest plot of **rs2241766** polymorphism and CAD under recessive comparison


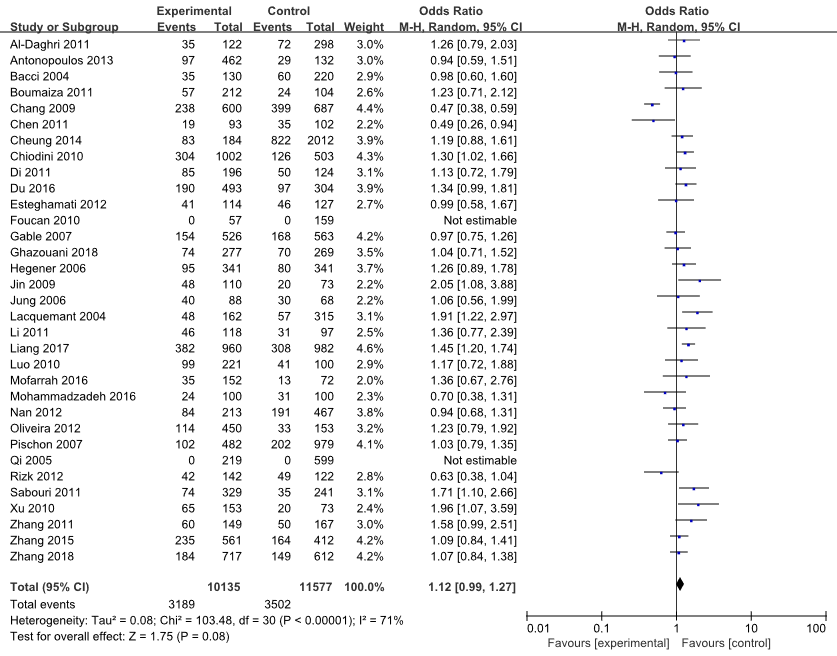


Forest plot of **rs2241766** polymorphism and CAD under over-dominant comparison


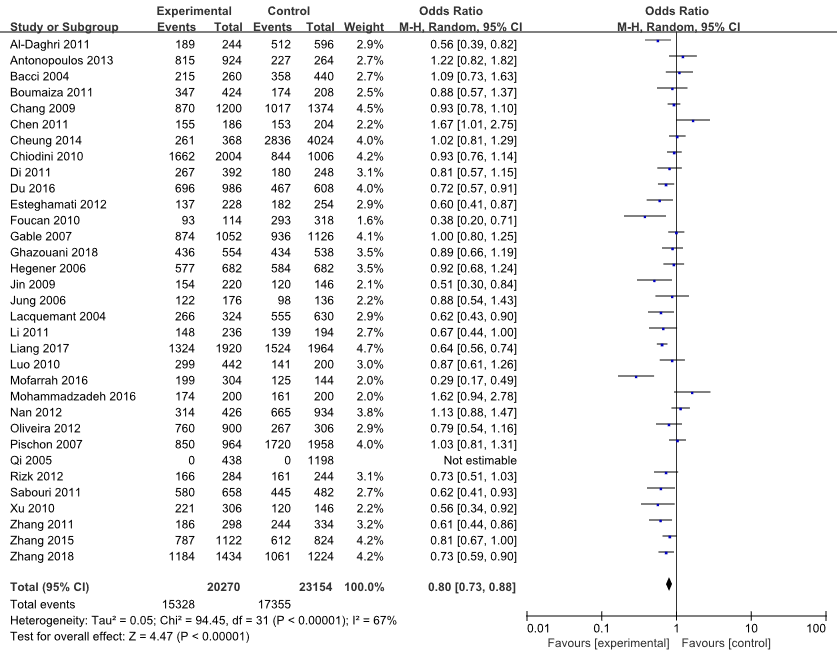


Forest plot of **rs2241766** polymorphism and CAD under allele comparison


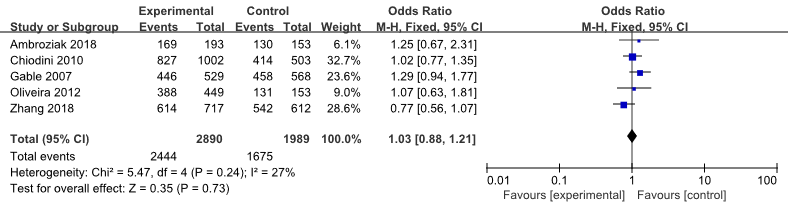
Forest plot of **rs17300539** polymorphism and CAD under dominant comparison

**
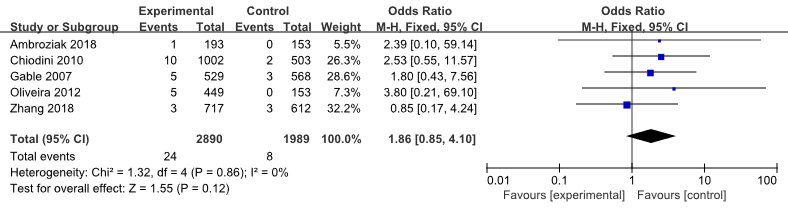
**

Forest plot of **rs17300539** polymorphism and CAD under recessive comparison


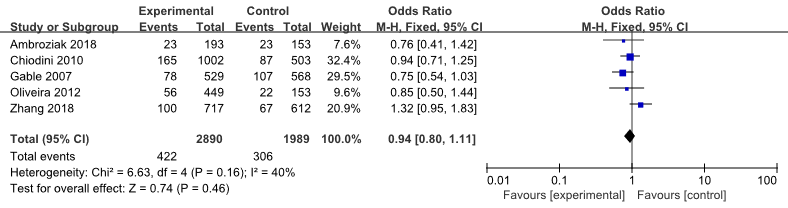


Forest plot of **rs17300539** polymorphism and CAD under over-dominant comparison


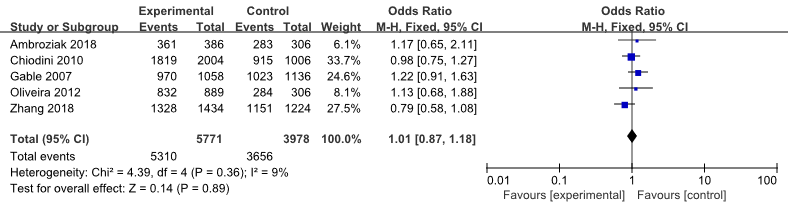


Forest plot of **rs17300539** polymorphism and CAD under allele comparison
